# Using Research to Transform Electronic Health Record Modernization: Advancing a VA Partnered Research Agenda to Increase Research Impacts

**DOI:** 10.1007/s11606-023-08289-y

**Published:** 2023-10-05

**Authors:** Alison M. Cogan, Seppo T. Rinne, Michael Weiner, Steven Simon, Jessica Davila, Elizabeth M. Yano

**Affiliations:** 1 Mrs. T. H. Chan Division of Occupational Science and Occupational Therapy, Herman Ostrow School of Dentistry, University of Southern California, Los Angeles, CA USA; 2 VA HSR&D Center for the Study of Healthcare Innovation, Implementation and Policy, VA Greater Los Angeles Healthcare System, Los Angeles, CA USA; 3 Center for Healthcare Organization and Implementation Research, VA Bedford Healthcare System, Bedford, MA USA; 4 Department of Medicine, Boston University Chobanian & Avedisian School of Medicine, Boston, MA USA; 5 Center for Health Information and Communication, U.S. Department of Veterans Affairs, Veterans Health Administration, Health Services Research and Development Service, Richard L. Roudebush VA Medical Center, Indianapolis, IN USA; 6 Department of Medicine, Indiana University School of Medicine, Indianapolis, IN USA; 7 Center for Health Services Research, Regenstrief Institute, Inc., Indianapolis, IN USA; 8 Department of Medicine, University of California, Los Angeles (UCLA) Geffen School of Medicine, Los Angeles, CA USA; 9 Center for Innovations in Quality, Effectiveness, and Safety, Michael E. DeBakey VA Medical Center, Houston, TX USA; 10 Department of Medicine, Baylor College of Medicine, Houston, TX USA; 11 Department of Health Policy & Management, UCLA Fielding School of Public Health, Los Angeles, CA USA

**Keywords:** electronic health records, informatics, Veterans health services, United States Department of Veterans Affairs

## Abstract

**Background:**

The U.S. Department of Veterans Affairs (VA) is undergoing an enterprise-wide transition from a homegrown electronic health record (EHR) system to a commercial off-the-shelf product. Because of the far-reaching effects of the EHR transformation through all aspects of the healthcare system, VA Health Services Research and Development identified a need to develop a research agenda that aligned with health system priorities so that work may inform evidence-based improvements in implementation processes and outcomes.

**Objective:**

The purpose of this paper is to report on the development of a research agenda designed to optimize the EHR transition processes and implementation outcomes in a large, national integrated delivery system.

**Design:**

We used a sequential mixed-methods approach (portfolio assessment, literature review) combined with multi-level stakeholder engagement approach that included research, informatics, and healthcare operations experts in EHR transitions in and outside the VA. Data from each stage were integrated iteratively to identify and prioritize key research areas within and across all stakeholder groups.

**Participants:**

VA informatics researchers, regional VA health system leaders, national VA program office leaders, and external informatics experts with EHR transition experience.

**Key Results:**

Through three rounds of stakeholder engagement, priority research topics were identified that focused on operations, user experience, patient safety, clinical outcomes, value realization, and informatics innovations.

**Conclusions:**

The resulting EHR-focused research agenda was designed to guide development and conduct of rigorous research evidence aimed at providing actionable results to address the needs of operations partners, clinicians, clinical staff, patients, and other stakeholders. Continued investment in research and evaluation from both research and operations divisions of VA will be critical to executing the research agenda, ensuring its salience and value to the health system and its end users, and ultimately realizing the promise of this EHR transition.

## INTRODUCTION

The U.S. Department of Veterans Affairs (VA) is undergoing an enterprise-wide transition from a homegrown electronic health record (EHR) system to a commercial off-the-shelf product. The stated goals of this transition are to improve VA providers’ ability to deliver high-quality health care for Veterans, and to promote interoperability with the U.S. Department of Defense (DoD) and community care providers, especially as VA increasingly pays for community-based non-VA care.^[1]^

The VA was among the earliest adopters of EHRs, with research and development of an EHR beginning in the 1970s, and nationwide implementation of the then ground-breaking Veterans Health Information Systems and Technology Architecture (VistA) and Computerized Patient Record System (CPRS) in the early 1990s.^[2–4]^ Developed by VA medical informaticists and clinicians, VistA/CPRS has long been recognized as being user-friendly for clinicians. Data generated in VistA/CPRS were used to create the VA Corporate Data Warehouse, which serves as a rich resource not only for computerized clinical decision support and management, but also for research and evaluation.^[5]^

Over the succeeding decades, efforts to upgrade and expand VistA to accommodate the needs of clinicians, researchers, and Veterans across the healthcare system have resulted in a complex system that is challenging to maintain.^[6]^ In 2017, VA decided to transition to a commercial off-the-shelf EHR product, and subsequently contracted with Cerner Corporation (now Oracle Cerner), enabling DoD interoperability given their transition to the same commercially-developed EHR.^[7]^ VA developed a 10-year phased implementation plan for a VA-adapted instance of Oracle Cerner’s Millennium EHR that began in October 2020, overseen by the VA Office of EHR Modernization (OEHRM; renamed as the EHR Modernization Integration Office [EHRM-IO] in 2022). The lifetime costs for the entire transition are estimated at over $50 billion and will affect virtually every facet of VA health care delivery, with implications for clinical care, quality, safety, research, education, and more.^[8]^

VA has a robust intramural research mission and commitment to quality improvement. A particular strength is VA’s model of embedded research, in which health service researchers are integrated across the healthcare system and partner with operations and clinical leaders to address real-world needs.^[9,10]^ Because of the far-reaching effects of the EHR transformation—affecting every clinical workflow for every clinical team member in over 1200 sites of care—research must be aligned with health system priorities enabling evidence-based improvements in implementation and implementation outcomes. Research must also be well coordinated to minimize burden on end-users; studying implementation requires collecting data from the same people who are learning the new EHR, all while continuing to deliver high-quality care for Veterans. The 10-year phased implementation plan for the new EHR also allows for research and evaluation findings from earlier deployment sites to systematically inform enhanced implementation strategies and outcomes for future deployments.

Despite the costs associated with EHR transitions and their implications for healthcare delivery, remarkably little is known about EHR transitions, their impacts, or outcomes. Published literature is scant and of variable quality, limiting our ability to apply rigorous evidence to future efforts.^[11,12]^ The need to accelerate EHR research to improve the process and outcomes of such transitions is critical, as is the science underlying their implementation. Although a national health information technology research agenda exists, it is necessarily broad to address many aspects of technology; developing a research agenda focused on EHR transitions could provide valuable guidance for investigators in this space.^[13]^ VA Health Services Research and Development (HSR&D) Service funded the Coordinating Hub to Promote Research Optimizing Veteran-centric EHR Networks (PROVEN) to provide a single point of contact for the VA research community to design, conduct, and disseminate cutting-edge, operationally prioritized research and evaluation to support safe and efficient implementation of the new EHR. One of the first challenges for PROVEN was the systematic development of a comprehensive research agenda. The purpose of this paper is to report on the process of developing the research agenda and its results.

## METHODS

### Approach to Agenda Development

**Fig. 1:**
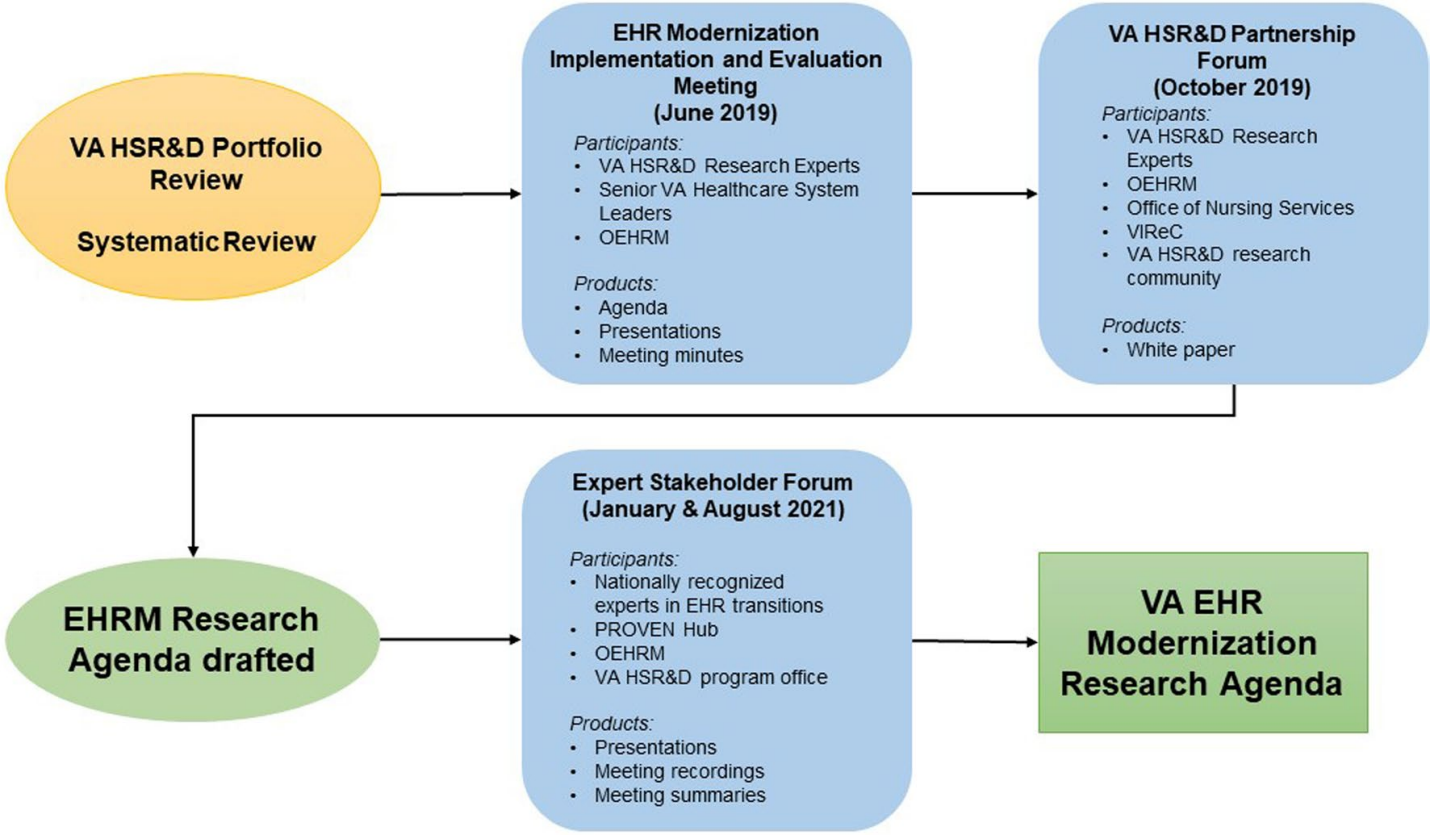
**Overview of steps to developing the VA electronic health record (EHR) research agenda. Abbreviations: EHR, electronic health record; EHRM, electronic health record modernization; HSR&D, Health Services Research and Development; OEHRM, Office of Electronic Health Record Modernization; PROVEN, Coordinating Hub to Promote Research Optimizing Veteran-centric EHR Research Networks; VA, Department of Veterans Affairs; VIReC, VA Information Resource Center.**

This agenda was developed using a sequential mixed methods approach to data collection and analysis. Using previous agenda development processes as a model,^[9,14–17]^ we (1) assessed the funded VA EHR-related research portfolio, (2) reviewed the published EHR transitions literature,^[18]^ and (3) convened stakeholder meetings with three key groups: VA researchers, VA operational partners, and external (non-VA) experts.^[19]^ These steps enabled us to reach consensus on a comprehensive EHR research agenda linked to researcher interests and capabilities linked to health system priorities and needs, anchored in nationally recognized EHR expertise to maximize rigor (Fig. 1).

#### Portfolio Assessment

We worked with the VA HSR&D scientific program manager overseeing VA health informatics–related research to identify all VA-funded studies related to EHR transitions. Despite VA’s longstanding EHR and embedded research workforce, replete with a large number of informatics-trained researchers and a dedicated scientific merit review board for informatics-focused proposals, only one funded study was identified (Miho Tanaka, PhD, personal communication).

#### Systematic Review

We collaborated with the VA Evidence Synthesis Program in the design and conduct of a rapid systematic review of published research or evaluation of EHR transitions in or outside the VA. Results demonstrated a dearth of VA-generated EHR-related published research and a modest literature outside the VA.^[18]^

### Stakeholder Engagement

#### Stage 1: EHR Modernization (EHRM) Implementation Evaluation and Research Meeting (Researcher-focused Agenda Development)

As implications of a new EHR for the research enterprise became clear, the VA HSR&D Service collaborated with field-based researchers (organized by EMY) to design and convene a day-long EHRM Implementation Evaluation and Research Meeting in Washington DC (June 2019). Approximately 25 participants attended including experts from the VA Office of Research and Development (which oversees HSR&D Service), representatives from OEHRM, and senior VA healthcare system leaders. Meeting objectives were to (1) identify areas where new research could provide the most value in improving implementation of the new EHR; (2) identify the most important outcomes to be assessed by research; (3) identify available data sources and possible study designs; and (4) discuss a timeline for soliciting and funding new research. The VA HSR&D Service Director, the VA HSR&D Scientific Program Manager for the informatics portfolio, and a VA HSR&D Center of Innovation Director (EMY) identified and invited VA researchers who had funding to conduct EHR-related research and/or were recognized as leaders in the field and with an eye to diverse geographic representation.

The meeting began with a welcome and brief opening remarks from senior VA research leaders (Assistant Under Secretary for Health for Discovery, Education, and Affiliate Networks [Clancy], Director of VA Office of Research & Development [Ramoni], Director of VA HSR&D Service [Atkins]). An OEHRM leader in change management [Dr. Robin Graff-Reed] provided an overview of OEHRM areas of concern and plans for change management to frame operations needs. Next, four presentations by VA research experts were delivered on frameworks for studying EHR implementation and transition (Hardeep Singh, MD, MPH, VA Houston and Baylor); research approaches from an informatics perspective (Michael Matheny, MD, MPH, VA Nashville and Vanderbilt); how implementation science can be helpful through the EHR transition (Amy Kilbourne, PhD); and an overview of ongoing primary care projects related to the anticipated EHR transition in the VA Puget Sound Healthcare System (WA) (Karin Nelson, MD, MPH, VA Puget Sound and University of Washington). These research presentations were followed by a discussion about issues of concern with the planned EHR, existing research on EHR implementation and design inside and outside the VA, and decisions that could benefit from VA research expertise, evidence, and contextual knowledge about VA healthcare delivery. Attendees then broke out into three concurrent discussion groups: (1) implementation, change management, and organizational structures; (2) patient and provider experience; and (3) impact on quality, safety, and other performance measures. Following the breakout sessions, the full group reconvened to debrief, summarize discussions, synthesis research topics, and identify next steps. Products that resulted from this meeting include the meeting agenda, list of attendees, presentations, and meeting minutes; these products were used as inputs to the design of the Partnership Forum in Stage 2.

#### Stage 2: VA HSR&D Partnership Forum (Engagement of VA Operational Partners)

In parallel, we (STR) actively engaged OEHRM and VA program office leaders (e.g., Office of Health Informatics, Office of Nursing Services) in the design of an evaluation of the initial “go live” sites in anticipation of launching a VA Quality Enhancement Research Initiative (QUERI) Partnered Evaluation Initiative. Through these efforts, several key operational partners for EHR-related research were identified, (including those with responsibilities for EHR change management, training, implementation and use), and invited to participate in a panel discussion at the national VA HSR&D/QUERI research conference (October 2019), a full year before the first site would launch the new EHR.

We (STR) organized a 3-h long partnership forum entitled, “Developing a Research Agenda for Cerner Implementation.” The forum’s primary objective was to collaboratively discuss EHRM research priorities, opportunities, and challenges with leaders from OEHRM, the VA Information Resource Center, and the Office of Nursing Services. The forum was open to all conference attendees; approximately 50 people participated. The session began with an overview of current EHRM research and partner perspectives on high-priority research areas. Panelists discussed cross-cutting themes for EHRM research including system implementation, system features to enhance quality and safety, increasing successful user adoption, provider behavior and well-being, and data collection and comparability for research and operations. Attendees broke out into four discussion groups, each tasked with identifying key research questions and prioritizing them according to partner goals and anticipated impact. All groups reconvened to summarize their recommendations and respond to questions. The forum concluded with reflections from VA operational partners and planning next steps, followed by post-forum development of a white paper summarizing discussion results (led by STR, reviewed by operational partners). We used outputs of Stages 2 and 3 to develop a draft research agenda.

One meeting outcome was recognition that to accelerate EHRM research effectively to keep pace with EHR deployment plans, a research coordinating hub would be needed. Within 6 months (April 2020), VA HSR&D had developed, fielded, and competed a Request for Applications to fund the PROVEN Hub.

#### Stage 3: External Stakeholder Panel (Validation of the Research Agenda with External Experts)

After drafting the research agenda, we then sought input on the draft from 12 external informatics experts as stakeholders, including 10 from outside VA. An additional two outside experts were invited but unable to participate. Experts were identified based on publications on EHR transitions, recognition in the field, and through snowball sampling.^[20]^ They represented over 10 diverse large healthcare systems and academic medical centers outside VA from current and past employment (e.g., Intermountain Health, Partners HealthCare System, Mayo Clinic, University of California) and served in a variety of senior leadership roles. Areas of expertise included health information technology leadership, EHR design and implementation, clinical informatics, EHRs and patient safety, EHRs and interoperability, and human–computer interface.

We convened external stakeholder panel meetings of these 12 experts in January and August 2021 via videoconference. Each meeting lasted 2 h and included PROVEN Hub leaders and project staff. Representatives from VA HSR&D and OEHRM attended as observers. In advance of each meeting, panelists were provided the draft research agenda and specific questions on which feedback was requested. A leading informaticist nationally recognized in and outside the VA (Hardeep Singh, MD, MPH) served as panel chair and discussion moderator. Prior to each meeting, he asked each attendee to be prepared to initiate discussion on one of the questions. After initial responses, conversation was open to input from all panelists. Participants also used the “chat” feature of the videoconferencing platform to share additional ideas and comments. Following each meeting, the PROVEN team prepared a summary of the discussion and distributed it to panel members. Products from these meetings included meeting agendas, presentations, meeting recordings, meeting chats, and summaries.

### Data Analysis and Synthesis

The authors developed a preliminary draft of the VA EHRM research agenda using results from the literature review and first two stages of stakeholder engagement. Summaries from each stakeholder engagement session were used to triangulate common themes and ideas, which were then refined and fed back to the External Stakeholder Panel for another round of review before finalization.

## RESULTS

We present the results of each of the three stakeholder engagement meetings, followed by the resulting research agenda. The systematic review included 40 publications about transitions from one EHR to another. The review identified gaps in patient safety outcomes, data migration strategies, capacity for health information exchange, care coordination, and user practices and workarounds; in addition, more methodological approaches to studying EHR transitions were recommended, including human factors, mixed methods, and implementation science. The full review is in press.^[18]^

### Stage 1: EHRM Implementation Evaluation and Research Meeting

Interactive discussion at the 2019 EHRM-focused meeting yielded several key questions across multiple domains. The group also raised pragmatic questions about the conduct of research after the EHR transition, including how researchers would access analyzable data, and strategies for rapid dissemination of research and evaluation findings. Breakout groups generated several potential research questions, summarized in Table 1. These questions informed the development of the partnership forum. For example, the research area of “Implementation, change management, and organizational structures” from stage 1 was used to frame the work of the “Implementation” breakout group in stage 2.

**Table 1:** Summary of EHR Research Agenda Topics Identified by Breakout Groups

Implementation, change management, and organizational structures	Patient and provider experience	Impact on quality, safety, and other performance measures
• What clinical and organizational factors facilitate adoption and use of new EHR systems? • How did the EHRM governance councils work? • What was the effectiveness of change management events?	• Which implementation strategies effectively engage clinicians in EHRM? • How do different multidisciplinary team members (physicians, nurses, trainees, etc.) experience EHRM? • How does EHRM impact clinician experience (workflow, efficiency, satisfaction, and burnout)? • How does the implementation impact patient experience?	• What is the safety impact on EHR sensitive care processes? • What types of workarounds occur within EHR implementation? • What is the impact on patient reported outcome measures? • What is the impact on routinely collected quality metrics?

*EHR* electronic health record, *EHRM* electronic health record modernization

### Stage 2: VA HSR&D Partnership Forum

In breakout sessions that included operations partners and VA HSR&D researchers, each of the four groups identified a set of potential research questions that were priorities for operations. Each operations partner identified research questions specific to their needs. Additional questions about quality of care were identified during cross-group discussions; key questions are summarized in Table 2.

**Table 2:** Key topics for partnered electronic health record-related research

Topic	Rationale	Example Research Questions
Implementation	Existing knowledge about best practices for implementation and change management is limited. Highest level EHRM leaders identified questions that would address knowledge gaps around successful end-user adoption of the new EHR.	• What implementation strategies and other factors influence the change management process? • What feedback systems are effective for improvement at the clinician and team-level? • How might the change process be measured at the pre- and post-level? How does it compare across sites? • How do changes made in one medical center trickle-down to effect employees in other medical centers when a standardized system is used? How can employees be efficiently re-trained on these changes?
Workforce	Office of Nursing Service identified research needs associated with how the transition to the new EHR would impact the nursing workforce as related to staffing needs, retention, and job roles.	• How does staffing levels and skill mix change and what are costs with such models that use recent patient needs to update staffing for the next day? • How does staffing, turnover, and vacancy rates influence implementation? • How do staff work around or adapt their work processes based on the new system? • How will employee job roles (scope of practice) change as part of the system implementation?
Quality of care	Multiple stakeholder groups identified research needs regarding the impact of the new EHR implementation on quality outcomes.	• What processes in the system effect patient care and patient interactions with staff? • How might the new EHR promote evidence-based practices and will this lead to quality of care improvements? • Because the system eliminates a need for manual data collection of several quality measures used for internal and public reporting, how will VA compare when using similar methods? Have quality measures been overestimated in the past? • What strategies are effective in managing productivity losses when transitioning to the new system? How long does reduced productivity last?
Data quality for research and evaluation	VA Information Resource Center is a key source of knowledge and guidance about using VA data for research. They identified key questions about the quality and usability of data from the new EHR for research and evaluation.	• How can metadata inform time to diagnosis, treatment, accurate diagnosis? • How can data between systems (current VA and new VA) be validated? What role do chart reviews or data-based metrics play? • How do patient records pre- and post-transition compare? • How can research involving multiple sites obtain comparable data when different systems are used?

*EHR* Electronic health record; *VA* Department of Veterans Affairs

The products from the first two stages were used to draft the research agenda. For example, the content that had been under “Implementation, change management, and organizational structures” from stage 1 and “Implementation” in stage 2 was synthesized under the “Operations” heading, which encompasses implementation, training, learning health systems, and organizational structures, along with EHR governance, data quality, and conduct of research.

### Stage 3: Expert Stakeholder Panel

Expert stakeholder panelists broadly endorsed the draft research agenda. They also identified high-priority research questions that would be impactful in the short term, related to VA research replicability using data from the new EHR, Veteran engagement in the EHR implementation process, human factors engineering, and organizational factors associated with EHR transitions. Given the unique opportunity of this large-scale transition, expert panelists recommended using pragmatic study designs, such as a multicenter, stepped wedge trial that could take advantage of the progressive EHR implementation across the VA. These recommendations were integrated into the research agenda. Panelists also made recommendations to develop infrastructure to expand and accelerate research on the EHR transition, including a specific funding portfolio and establishment of learning labs. Similar to discussions in the preceding HSR&D meetings, they emphasized the need for agile research that was responsive to the short-term needs of operational partners.

### VA EHR Modernization Research Agenda

Table 3 summarizes the research agenda. Research questions focused around operations, user experience, patient safety, clinical outcomes, value realization, and informatics.

**Table 3:** VA EHRM research agenda

Topic	Example Question
Operations	
Conduct of research	How is research affected, what are the advantages, and how can challenges be met?
Data quality	How can data between VistA/CPRS and the new EHR be validated?
EHR governance	How have EHR governance structures influenced the ability to scale and spread EHR-based innovations?
Implementation	What feedback systems are effective for improvement at the clinician and team-level?
Learning health system	How is EHRM affecting VA as a Learning Health System?
Organizational factors	What organizational factors facilitate adoption and use of the new system?
Training	Which training methods are most effective and sustainable, considering the periodic turnover of clinical staff and trainees?
User experience	
Access to care	How has appointment scheduling changed and what is its impact on wait times for appointments?
Efficiency/time use	How much time do healthcare providers spend in direct patient care vs. EHR-related tasks (documentation, ordering, etc.)?
Electronic professional communication	How are professionals interacting with each other via the EHR, inside and outside VA, in teamwork and consultations?
Patient portal	Does the patient portal provide information that Veterans need in a way they can understand it?
Provider workflow/job role changes	How will employee job roles (scope of practice) change as part of the system implementation?
Veteran experience	How does the EHR transition impact Veterans’ experiences of care?
Patient safety	
Health information exchange and integrations	To what extent is EHRM supporting the need for health information exchange and interoperability of systems inside and outside VA?
Quality of care	How does using the EHR affect the quality and safety of medical care?
Workarounds	How do staff work around or adapt their work processes based on the new system?
Visualizations, alerts, and decision support	How have visualization of data, alerts, and medical decision-making, changed with EHR modernization, and what are the outcomes?
Clinical outcomes	
Patient-reported outcome measures	What is the impact on patient reported outcome measures?
Quality metrics and reporting	What is the impact on routinely collected quality metrics?
Value realization	
Burnout	How does staffing, turnover, and vacancy rates influence implementation?
Cost and value of care	How does EHRM affect the role of the EHR in assessing and improving the cost and value of medical care?
Staffing needs	How does staffing levels and skill mix change and what are costs with such models that use recent patient needs to update staffing for the next day?
Informatics	
Human factors	How do issues of human factors and human–computer interaction relate to patient safety, burnout, cognitive load, and cognitive function?
Informatics workforce	What organizational practices are most effective at transforming the VA’s informatics workforce to meet the new EHR environment?
Information foraging	How does the modernization reduce burden on clinicians to search for information about common medical conditions?
Technological innovation	What is the effect of EHRM on innovation, and capacity for innovation, with the EHR?

*CPRS* Computerized Patient Record System, *EHR* electronic health record, *EHRM* electronic health record modernization, *VistA* Veterans Health Information Systems and Technology Architecture

## DISCUSSION

With well over 200,000 healthcare personnel at nearly 1300 facilities, the VA transition from VistA/CPRS to a new EHR is among the largest EHR-to-EHR transitions attempted. Its occurrence in a national integrated delivery system with embedded research infrastructure has created unprecedented opportunities to conduct research and evaluation on all aspects of the EHR transition. Using a multi-level stakeholder engagement process, we developed a comprehensive EHR-focused research agenda to identify high-priority, operationally aligned research topics. The PROVEN Hub was funded early on to coordinate research and evaluation, prevent duplicative work, and limit burden on frontline staff faced with fundamental changes to their clinical and administrative workflows. The resulting agenda includes a broad range of topics that encompass both pragmatic operational issues and fundamental changes to healthcare delivery across VA, increasing EHR-focused granularity compared to broad informatics research agendas. Carrying out this agenda will necessitate expansion of the VA informatics research workforce, funding mechanisms to support rapid-pace research and evaluation, and ready access to needed data.

There are numerous challenges to implementing this research agenda. Not surprisingly, the new EHR is undergoing changes with each implementation wave to increase capabilities and correct problems; thus, the tool itself is evolving. Different data structures and clinical workflows between old and new EHRs also hinder comparative research between VistA/CPRS and Cerner sites. Implementation of the new EHR also began in October 2020, with front-line staff as primary end users already overtaxed from months of providing healthcare in the context of the COVID-19 pandemic.^[21–23]^ EHRM leadership and governance—key to effective transitions—have also undergone many changes, necessitating continuous relationship building between research and operations leaders. To that end, PROVEN continues to engage with EHRM-IO, numerous clinical program offices, local and regional operations leaders, and the research community. Patient safety events and other problems have been documented by the VA Office of the Inspector General.^[24,25]^ Program leaders have been called to multiple hearings before the US House of Representatives and Senate committees that have oversight responsibility for the EHR’s modernization.^[8,26]^ Problems were also identified with end-user training effectiveness.^[27]^ And, the VA EHR transition represents a cultural transformation toward national standardization of procedures and documentation across diverse healthcare facilities. Collectively contributing to implementation delays, these and other challenges have complicated the advancement of EHR-focused research.

Nonetheless, research and evaluation have progressed in many areas. VA QUERI has supported two partnered evaluation initiatives related to the EHR transition. The first is a mixed-methods evaluation that is designed to understand clinician and staff experiences with the EHR transition with a goal of identifying challenges and promoting improvement opportunities for successive waves of the transition. The second focuses on the needs of health professions trainees, who play a critical role in care delivery across the VA, and will assess specific solutions to improve their experience with the EHR transition. Through the PROVEN Hub, VA HSR&D has supported ten pilot projects addressing a range of research areas including patient safety, referrals management, governance, organizational readiness for change, women’s health, data quality, and Veteran experience. PROVEN also facilitates a Patient Safety and Human Factors Research Workgroup to help researchers collaboratively develop new projects. VA HSR&D recently funded a new study focused on employee burnout related to the EHR transition.

There are other approaches to developing a research agenda;^[28,29]^ using a different set of methods may have resulted in different priorities. Our portfolio review was also limited to active projects funded by VA HSR&D and QUERI. We may have missed projects funded by other agencies.

## CONCLUSION

The VA’s transition to a new EHR presents an unprecedented opportunity to pursue research capable of improving implementation and outcomes. This research agenda was designed to guide development of future science aimed at generating actionable results to address the needs of operations partners, clinicians, patients, and other stakeholders. The VA has already made substantial investments in supporting this area of research. Continued investment in research and evaluation from both research and operations divisions of VA will be critical to executing the research agenda, ensuring its salience and value to the health system and its end users, and ultimately realizing the promise of this EHR transition.
